# Identifying Functional Mechanisms in Psychotherapy: A Scoping Systematic Review

**DOI:** 10.3389/fpsyt.2020.00291

**Published:** 2020-04-17

**Authors:** Timothy A. Carey, Robert Griffiths, James E. Dixon, Sonia Hines

**Affiliations:** ^1^ Institute of Global Health Equity Research, University of Global Health Equity, Kigali, Rwanda; ^2^ Mental Health Nursing Research Unit, Greater Manchester Mental Health NHS Foundation Trust, Manchester, United Kingdom; ^3^ Division of Nursing, Midwifery and Social Work, School of Health Sciences, Faculty of Biology, Medicine and Health, University of Manchester, Manchester, United Kingdom; ^4^ North West Boroughs Healthcare NHS Foundation Trust, Warrington, United Kingdom; ^5^ Centre for Remote Health, Flinders University, Alice Springs, NT, Australia

**Keywords:** mechanisms, psychotherapy, functional, statistical, mediators, neuroscience, change processes, effectiveness

## Abstract

The identification of fundamental mechanisms is an important scientific pursuit in many fields of enquiry. With regard to the development of psychological treatments, understanding the mechanisms through which change occurs such that psychological distress resolves, can enable us to develop more effective and efficient interventions. In the field of psychotherapy, mechanisms are often identified either statistically or conceptually. The most powerful and useful mechanisms, however, are functional rather than statistical or conceptual. More specifically, with regard to mechanisms relevant to psychotherapy, it is difficult to identify what any of these mechanisms actually *do* in a mechanistic sense. That is, the mechanics of putative mechanisms are generally unspecified. In order to obtain a rigorous and comprehensive account of the current mechanisms in psychotherapy, as well as to evaluate their usefulness, a systematic scoping review was conducted. The systematic scoping review did not yield any mechanisms that were expressed in functional terms. We argue that, in order for psychotherapy to improve its effectiveness and efficiency, the standard for what is accepted as a useful mechanism needs to be substantially raised. Only functional mechanisms that express plausible actions consistent with known biological processes should be used to inform therapeutic interventions.

## Introduction

In the field of psychotherapy, the term “mechanism” refers to an explanation of how psychotherapeutic interventions translate into events that lead to the desired outcome (1). Kazdin defines a mechanism as “the basis for the effect, i.e., the processes or events that are responsible for the change; the reasons why change occurred or how change came about.” (1) (p. 3). As we will explain below, for this systematic review we have included a broad search strategy, however, it is perhaps uncontentious to suggest that functional, physical mechanisms such as negative feedback are more robust and, ultimately, more useful for scientific progression than nonfunctional, conceptual mechanisms such as “fear-of-fear.” Despite the existence of a plethora of approaches to psychotherapy—with several hundred distinct psychotherapeutic models or techniques described to date (2) —it is still not clear exactly how or why these approaches produce their effects (3).

### The Importance of Understanding Mechanisms

Understanding how and why psychological treatments work is important for a number of reasons. Firstly, although there is evidence that psychotherapy is helpful for many people who report psychological distress, there is considerable variation between individuals in terms of the amount of change experienced as a result of engaging with therapy (4). Part of this variation no doubt reflects the different ways in which change is defined such as by differences in scores on outcomes measures or reduction or increases in identified behaviors. Indeed, for some clients, engaging with psychotherapy actually seems to lead to negative outcomes such as a deterioration in functioning (5). Greater understanding of the mechanisms of change would help to clarify which clients are likely to gain the most benefit from therapy and under which conditions. Secondly, a better understanding of mechanisms would help to close the theory-practice gap that has been identified as an impediment to the implementation of evidence-based psychological treatments (4, 6, 7). More broadly however, there remains a lack of understanding holistically the way in which psychological effects and biological mechanisms relate and emerge from one another (8). Bridging these gaps will be necessary to move the development of psychotherapies further. As important and powerful as our psychological processes are, we cannot escape our biology. Any model or process that is proposed must be consistent with known biological structures and properties. Substantial progress will only be made when models are developed that genuinely articulate bio psycho social functioning (8). Thirdly, at present, we do not understand whether there is a single mechanism through which psychotherapy effects change, or whether there are actually multiple mechanisms involved. This is an important distinction to make in terms of promoting the most effective and efficient methods to assist in the alleviation of psychological distress. If there is one fundamental mechanism of change then efforts need to focus on how to access and harness this mechanism. If, however, there are multiple mechanisms of change, a number of other decisions might be important such as selecting the mechanism to focus on or investigating whether multiple mechanisms need to be activated simultaneously or in a particular sequence. A more sophisticated understanding of mechanisms, therefore, would contribute to the development of psychological therapies that facilitate change in the most efficient and effective way possible.

Research examining the effects of psychotherapy on different populations has often observed that some people in the control groups show greater improvement than people in the treatment group. The design of such trials, however, means that researchers tend not to focus on the fact that some people who received no treatment will improve more than some people who received the active treatment. Aggregating data and emphasizing instead, central tendencies between the groups, masks this result (9). In fact, Blampied argues that, if we are to create a science of individuals, statistics is, fundamentally, the wrong approach, because the direction of inference in statistics is always from the sample to the population (10). This is exactly the opposite direction that is needed if we are to understand how individuals function. We will never discover fundamental properties of individuals by continuing to accumulate and assess aggregate data. Accordingly, Bolles asserts that, wherever possible, one should avoid statistics, “abolish superfluous rituals and routines, and get on with the business of science” (11) (p. 79). Of relevance here is the observation that “the power and precision of the natural sciences arose because of a focus on invariance or the common, fundamental underlying properties of seemingly distinct objects” (12) (p. 128). Increasing the extent to which programs of research build functional models to test fundamental assumptions by comparing data generated by the model with the data being investigated rather than relying almost exclusively on the accumulation of statistical success in the form of p values of a specified magnitude might begin to move the field in the direction that Blampied envisaged (10). This should not be construed as either/or a debate but rather a matter of balance. Inferential and descriptive statistics are extremely useful in identifying areas for further investigation. A thorough understanding of these areas, however, should be sought by the building and testing of functional models.

### How Mechanisms Have Been Defined in Other Fields

To illustrate how the practice of psychotherapy might be improved by the development of a robust mechanistic account of the change process, it is worth considering how other fields have approached this issue. In the field of medicine, for example, aspirin is a commonly used analgesic and antiinflammatory compound. The mechanism through which aspirin reduces pain and inflammation is by inhibiting the production of an enzyme called cyclooxygenase that stimulates the formation of prostaglandins, lipid compounds known to cause inflammation. An unintended consequence of taking aspirin, however, is that it prevents the production of prostaglandins which are important for the health of the stomach and kidneys (13). It is important, therefore, to understand aspirin’s effects at a biological level in order to gain the maximum benefit from its use. Although our understanding of aspirin’s mechanism of action is relatively advanced, it is certainly not the case that mechanisms are fully understood for all prescribed drugs. The danger of progressing to clinical trials without a clear understanding of a drug’s mechanism of action, however, can lead to expensive failures in the late stages of testing and place patients at risk of side effects that are hard to predict. For this reason, it is recommended that researchers who are using clinical trials to evaluate complex interventions understand “…how the intervention works: What are the active ingredients and how are they exerting their effect?” (14) (p. 1-2). We would add that understanding the active ingredients requires a sound understanding of known biological processes.

In fact, many of the early psychotropic drugs were discovered serendipitously with no clear explanation for their purported mechanisms of action (15). This situation allowed researchers to propose arguments that were seriously flawed in their reasoning (16, 17). For example, it was proposed that, because some medication increased people’s levels of serotonin with consequent elevations of mood being observed, then depressed mood must be caused by a serotonin deficiency (18). Once the chemical imbalance hypothesis was introduced it became impossible to remove despite their never being any evidence to support it (18, 19). So, by proposing an explanation that was not linked to any established biological processes, damaging effects have occurred such as the disabling of known homeostatic mechanisms in the brain and an increase in the number of neurotransmitter receptors leading to long-term dependency (19). Once again, the importance of understanding and integrating biological with psychological and social processes is demonstrated.

Having a sound understanding of aspirin’s mechanism of action, however, means that it is possible to understand both the intended effects and possible side effects of its use. It also means that the drug can be targeted to treat the people who are most likely to benefit from receiving it. A similarly robust explanation of the mechanisms through which psychotherapy leads to the amelioration of psychological distress would guide future research and support the development of more effective and efficient psychotherapeutic practices.

### Putative Psychotherapeutic Mechanisms

A large number of candidate mechanisms have been proposed to explain how psychotherapy exerts an effect. The examples we provide here in no way represent an exhaustive list. A recent meta-analysis of mindfulness-based cognitive therapy for depression, for example, supported the view that therapeutic effects were achieved through alterations to mindfulness, rumination, worry, compassion, or meta-awareness (20). A review of mechanisms in cognitive therapy for depression, however, pointed to the role of cognitive mediation – specifically, changes in “depressogenic schema” (21). A study of cognitive behavioral therapy (CBT) of panic disorder concluded that the mechanisms of change were increased self-efficacy and reduced anxiety sensitivity (22). Another study, however, proposed that CBT for panic disorder achieved its effect through a reduction in “fear-of-fear”; in other words, the tendency to respond fearfully to altered bodily sensations associated with anxiety (23). What can we conclude from the fact that the field of putative psychotherapeutic mechanisms is so diverse? One conclusion could be that different psychotherapeutic orientations and techniques have different mechanisms of action. Currently, there are many apparently different therapies with different methodological frameworks. The extent to which these superficial differences reflect differences in fundamental mechanisms of action, however, is far from clear. Another related conclusion could be that psychotherapy achieves its effects through different mechanisms depending on the nature of the disorder being treated or the diagnosis of the person seeking therapy. Also, a problem that plagues the field in the absence of a functional mechanism is being able to separate a mechanism from the *outcome* or effects of the mechanism. For instance, should increase self-efficacy and reduced anxiety be considered mechanisms of change or the *products* of some mechanistic process?

To increase our capacity to explain how psychotherapy works, efforts have been made to draw from the growing field of neuroscience, using data showing correlations between biological systems (e.g. fMRI scans of activated brain regions) with psychological processes (e.g. the self-reported experience of emotion), (for an example see Cozolino, 2010) (24). Causal inferences are then made between the two. Many of those, however, who have attempted to use this method to explain outcomes in psychotherapy have been forced to concede that examining two events sharing a moment in time (i.e. an experience reported by a person and a corresponding image of the brain metabolizing glucose) is insufficient when trying to explain how each phenomena contributes to the formation of the other (25). Hence, Johansson and Høglend have been led to conclude that: “…no definitive mechanisms of change for any type of psychotherapy have been satisfactorily demonstrated” (26) (p. 8). This view is shared by Kazdin, who argues that “After decades of psychotherapy research and thousands of studies, there is no evidence-based explanation of how or why even the most well-studied interventions produce change, that is, the mechanisms through which treatments operate” (3) (p. 148). Despite the current lack of progress in this area we fully endorse and support efforts to integrate biological with psychological and social processes. Perhaps what is needed is a different approach as to *how* this integration is investigated.

### Functional and Conceptual Models

One limitation of mechanisms of psychotherapeutic change proposed to date is that they are generally conceptual rather than functional in nature. The similarities and differences between putative mechanisms outlined in conceptual models can be hard to discern because of the inherent imprecision and potential for ambiguity that arises from the fact that these models are described in purely linguistic terms. As we have seen, reduced “anxiety sensitivity” and “fear-of-fear” have both been proposed as the mechanism of change for CBT for panic disorder (22, 23). Superficially, anxiety sensitivity and fear-of-fear might appear to relate to similar processes, even if they are described using different terminology. Because these two mechanisms are described in purely conceptual terms, however, it is hard to know to what extent they are truly distinct versus overlapping. The issue raised previously is also relevant here in that it can be hard to discern whether a reduced fear-of-fear is a mechanism of change or the result of the workings of a change mechanism.

Conversely, putative mechanisms described by functional models have the advantage that they are expressed in precise mathematical terms. The use of functional models means that potential ambiguity about the nature of the phenomena being described is reduced (27). When functional models are compared with each other, therefore, it is possible to have greater confidence that they are describing either the same or different phenomena. Recognition of the importance of precise, quantitative models to scientific endeavors is far from being a new idea. Guilford suggested that “the progress and maturity of a science are often judged by the extent to which it has succeeded in the use of mathematics” (28) (p. 1). Much earlier still, Thomson, said in a lecture “When you can measure what you are speaking about, and express it in numbers, you know something about it; but when you cannot measure it, when you cannot express it in numbers, your knowledge is of a meager and unsatisfactory kind: it may be the beginning of knowledge, but you have scarcely, in your thoughts, advanced to the stage of *science*” (29) (p. 73).

The history of medical and psychological sciences is replete with theories that aimed to explain the etiology and amelioration of psychological distress, which were considered plausible at the time but have since been disproved. For example, Hippocrates deemed human health (or the idea of equilibrium) as the harmonious balance of four vital humors that governed physiology and mood, a mechanistic theory that persisted for hundreds of years right up to the Medieval and Renaissance eras (30). Problems of health were believed to arise when the balance between these essential humors was lost. For example, melancholy was believed to be caused by an overabundance of black bile (of Greek origins—mela, meaning black, and chole, meaning bile) (31). A similar story arises from another Greek influence which suggested that a wandering womb was the source of anxiety in women—later influencing the descriptor of such conditions as hysteria (hysterikos—Greek meaning from the womb) (31). The familiarity of this language to us today testifies to a tendency to accept mechanistic theories based on purely conceptual descriptions before confirmation can be achieved through functional evaluations of the concepts.

It is our assertion that progress in psychotherapy, in terms of improving its effectiveness and efficiency, will be realized by emphasizing functional models and paying less attention to purely conceptual or statistical models or models that do not seem to relate in any way to known brain processes. In order to obtain a rigorous and comprehensive account of the current mechanisms in psychotherapy, as well as to evaluate their usefulness, a systematic scoping review was conducted.

### Review Question

What mechanisms used to account for psychological change in psychotherapy are supported by neurological or biological evidence and are expressed in functional terms?

## Methods

We used the Joanna Briggs Institute methods for scoping reviews of evidence to guide the conduct of this review (32). While a protocol was not registered, one was developed by the authors *a priori* to select the methods and criteria for inclusion before the review was begun.

### Inclusion Criteria

#### Population

The population of interest to this review were adults, with no other restrictions on demography. Studies describing their population as children, adolescents, or pediatric were excluded since we were interested in investigating the change process in a fully developed human brain not in one where the change process might be difficult to disentangle from standard developmental processes.

#### Concept

The concept of interest was the biological function or process underpinning psychotherapeutic change mechanisms. Eligible studies needed to identify both a change mechanism and a related or underlying biological function or process to be included. A change mechanism was defined as “a specific process through which thoughts, feelings, and behaviors, or some combination of these, was altered” and biological functions or processes were defined as “widely accepted brain activity such as synaptic transmission or signal propagation”.

#### Context

The context of interest was the nexus between neurobiological science and adult psychotherapy.

#### Types of Studies

Any type of quantitative research study or systematic review that was eligible for inclusion provided it reported evidence of plausible biological functions or processes associated with psychotherapeutic mechanisms of change was eligible for inclusion. Studies published after 2000 in English were eligible for inclusion. Our reasoning for beginning our search with publications from the year 2000 was that this would provide us with two decades of research to scrutinize and would also provide us with a decade of research both before and after Kazdin’s comments about the state of our knowledge in this area (3).

#### Exclusion Criteria

Animal studies, pediatric studies, discussion and opinion papers, studies of the mechanisms or functions of psychoactive medications or other drugs, and studies discussing or proposing mechanisms conceptually or statistically were ineligible for inclusion.

### Searches

We searched Medline, PsycINFO, EMBASE, and Google Scholar in May 2019 for research studies and systematic reviews meeting the inclusion criteria. The reference lists of retrieved articles were also screened for potentially relevant articles.

#### Search strategy

We used combinations of keywords and subject headings to construct search strategies appropriate for each of the databases. Initial keywords were psychotherapy or psychotherapeutic, change mechanism or mechanism of change or mechanism and biological or physical or function. Searches were downloaded from the databases into Endnote X9 (Clarivate Analytics, PA).

The titles and abstracts (where available) of search results were initially screened by two authors working independently to assess the congruence of studies to the inclusion criteria and identify papers to be retrieved in full text. Full text articles were then retrieved and screened independently by two authors to determine final inclusion status.

### Data Extraction

We planned for one author to extract data from the included studies. Units of extraction were citation details, study design, setting, and population where available, and details of the psychotherapeutic change mechanism with the related functional, neurological, or biological supporting evidence. It was also arranged that a second author would check the extractions against the papers of any included studies.

### Data Synthesis

We planned to use graphs and tables to synthesize the findings of all included studies.

## Results

**Figure 1:**
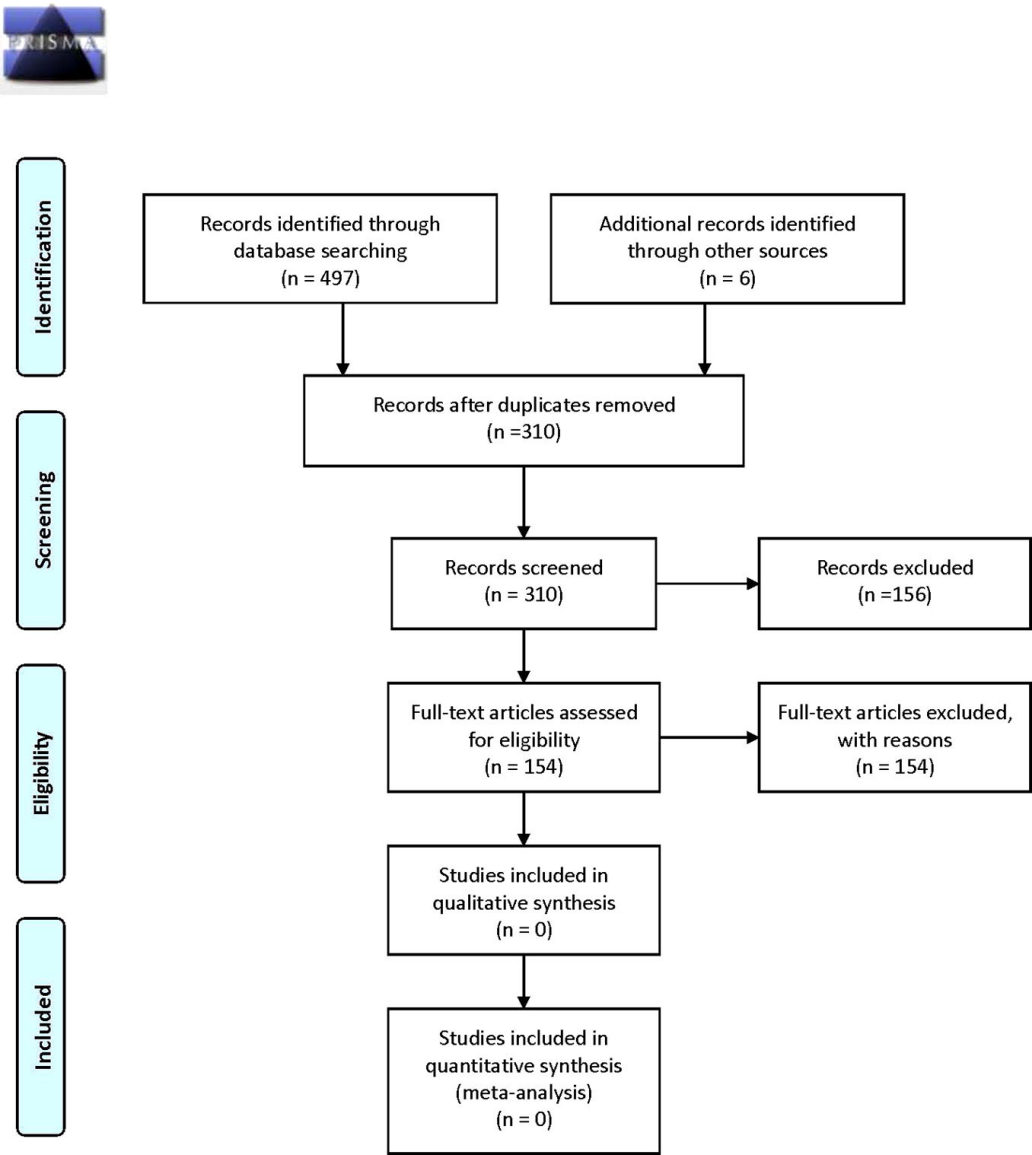
PRISMA 2009 flow diagram.

Searching identified 497 potentially relevant citations, with six further papers uncovered from reference list checking for a total of 503 papers for initial consideration. Of the potentially relevant citations, 154 were deemed likely to meet the inclusion criteria and retrieved in full text form. After reading the full text, no studies were retained to inform the analysis as none completely met the inclusion criteria. The flow of studies through the review process is illustrated in **Figure 1**.

Of the 154 articles selected for full-text examination, 70 were excluded for not being primary research, 66 for the research not examining functional mechanisms, 10 were systematic reviews, 2 focused on pediatric publications, 2 were not quantitative research, 2 were drug studies, 1 was a study protocol, and 1 article had been withdrawn by the publisher. Reasons for exclusion are further detailed in Appendix A in **Supplementary Material**.

## Discussion

In order to more accurately understand the change process in psychotherapeutic treatments, a scoping systematic review was conducted to identify mechanisms that were expressed in functional terms and that had some connection to known neurobiological brain processes. The literature search conducted as part of this scoping review returned numerous putative mechanisms that have been proposed as explanations for how change occurs as a result of engaging with psychotherapy. None of these mechanisms, however, were expressed in functional terms and none were related to known neurological or biological processes. The fact that our search strategy returned no studies for inclusion does not mean that the literature in this field is devoid of useful information. It does mean, however, that there are no current published studies that are able to answer our research question. It also means that a different approach to the consideration and study of mechanisms is further justified.

The majority of mechanisms identified in the process of this scoping review were described in purely conceptual or statistical terms. Whereas functional models aim to describe, in precise mathematical terms, the properties of systems comprising multiple interacting subsystems that are responsible for producing the phenomena of interest, conceptual models have been criticized for their reliance on abstract generalizations (33). The fact that conceptual models are expressed in verbal rather than mathematical terms has also been highlighted as a limitation. Models expressed in purely linguistic terms are inherently ambiguous and susceptible to misinterpretation (27). A consequence of this lack of specificity is that conceptual models are of limited use when trying to describe the inner organization of complex systems that are producing observable behaviour (33).

A proportion of putative mechanisms identified during the review process took the form of descriptions of changes in neural activity in particular areas of the brain (eg. Lueken et al., 2013; Messina, Sambin, Palmieri & Viviani, 2016; Reinhardt, et al., 2010) (34–36). It was not clear, however, how these changes in neural activity constituted a mechanism of change.

The findings of this scoping review are consistent with much of the existing literature on the topic of psychotherapeutic mechanisms, where the lack of plausible mechanisms of change have been identified as a barrier to developing more effective approaches to psychotherapy (1, 3, 26). Indeed, despite the fact that more than a decade has passed since Kazdin published a well cited article outlining this problem, it is disheartening to find that the field of psychotherapy research appears to be no closer to identifying such mechanisms (1).

Although Kazdin’s proposed framework for defining mechanisms within psychotherapy using a statistical process has been available for over a decade, not one study was able to meet all the requirements necessary to support a mechanistic conclusion (1). To compound this issue, not one study that we reviewed acknowledged these omissions as a limitation nor held back from making mechanistic inferences.

If we are to acknowledge this fully within the field of psychotherapeutic research, it leads to a possible crossroads in the development in this science. Do we repeat the last ten years and seek to improve the statistical processes used to discover mechanisms or do we do something different? Carey suggested in a review of the way in which psychotherapy creates its effects, that the process of reorganization might be a plausible change mechanism to explain the amelioration of psychological distress generally (37). The model of reorganization suggested by Carey is expressed as a functional model and is consistent with recognized neural processes (33, 37). Furthermore, the reorganization model can account for the nonlinear and unpredictable nature of the change process (38). A model such as this could lead the way to a new and, ultimately, more productive area of research. For progress such as this to occur, however, there needs to be a much stronger link between robust theories and research and clinical practices. That is, both researchers and clinicians working in the psychotherapy field should be required to link their practices to rigorous scientific theories incorporating functional mechanisms and established biological processes.

Kazdin has provided a coherent template for researchers aiming to identify mechanisms of change using statistical methods. This template, however, does not appear to have resulted in increased knowledge about mechanisms (1). It might be the case that were Kazdin’s recommendations for mechanistic research implemented rigorously—something that is not happening at present—it might result in the identification of plausible mechanisms (1). Recently, however, there have been calls for changes to evaluation practices to help improve the effectiveness and efficiency of psychological interventions, and to increase progress toward identifying plausible mechanisms of psychotherapeutic change (27, 39, 40). These changes would involve the adoption of a different approach to research practices to the one proposed by Kazdin (1).

Concerns have been expressed about the impact of implicit but frequently unstated assumptions that underpin the research designs of studies in this area (39). One such assumption is that of *linear causality*: the belief that an independent variable (the treatment, intervention, or technique) *causes* changes to the dependent variable (the outcome being measured). This assumption, however, fails to acknowledge that, in the case of psychotherapy, change does not happen independently of the client and therapist. In such circumstances, the treatment does not have any inherent therapeutic properties. Rather, therapeutic change arises from the interaction between therapist and client (41). One consequence of this assumption appears to be that many researchers have moved to testing psychological interventions, through the use of research designs such as randomized controlled trials (RCTs), without first conducting mechanistic research that could inform the design of such studies. Our position is that substantial progress will be made when research of functional mechanisms is embraced on a much wider scale and incorporated into research programs using designs other than RCTs to explore areas such as the way in which the therapist and the client co-create beneficial outcomes.

Given the current large-scale financial investment in psychotherapy research and practice, the fact that we were unable to identify any mechanisms meeting the criteria of this scoping review is concerning. In the United Kingdom alone, the *Increasing Access to Psychological Therapies* (IAPT) program is estimated to have cost £1 billion to date (42). For ongoing large-scale investment to be justifiable, it is not sufficient for researchers to identify that a relationship exists between attending therapy and improved outcomes. For the field to advance, and for therapists to become more helpful for more people more of the time, it is imperative that we improve our understanding of *how* and *why* engaging with psychological therapy translates into positive outcomes for service users. This will only occur when researchers and clinicians work together in the embrace of scientific theories that have established the role of functional mechanisms through rigorous testing. Only in this way will it be possible to understand which of the many available therapies will be most helpful, under what conditions, and to which people.

### Lack of Evidence

The current review of the field, using Kazdin’s framework to assess the quality of mechanistic inferences made within psychotherapy research, also highlights further barriers to explaining why and how psychotherapy works (1). It is our position that, even if Kazdin’s framework were imposed faithfully, the field is still far short of being able to define mechanisms involved in psychotherapeutic change. A purely statistical approach, we argue, will be insufficient. Looking to achieve the specificity called for by Kazdin, for example, will not be achieved through statistical modeling alone. Statistical models can examine the relationships between concepts, but statistics alone cannot attest to the ecological validity of the concepts being analyzed. In addition, the “meticulous detail” called for by Kazdin, again, cannot be provided through statistical modeling in isolation. Functional examples, working in the real world, are required to genuinely test whether a purported mechanism acts in a mechanistic way.

Again, looking back to history and the evolution of the scientific method suggests the current field researching psychotherapeutic mechanisms is struggling with fundamental epistemological issues. Popper has highlighted that providing incremental information does not necessarily increase knowledge, and warned strongly against using statistical inference alone (43). Further argued by Taleb who highlighted such limitations using the “Black Swan” example attributed to Mill. Hume also called out examples of naive empiricism, showing how induction becomes a problem if applied alongside an incorrect method, such as searching for a mechanism without the correct experimental design (44, 45). Looking for how someone achieves change through a therapeutic process requires the method to capture the adaptation, reorganization, and emergent factors specific to that person. Induction acts against such a focus, minimizing the variance and compressing the detail so as to lose the specific in favor of the general. Aside from these epistemological issues, there appears to be a problem with the applied assumptions and how the problem of mechanisms is being framed. Although we were not able to identify any studies that satisfied the inclusion criteria for this systematic review, we are, nevertheless, able to make the following conclusions:

1. Mechanisms in the psychotherapy field are discussed almost exclusively in conceptual or statistical terms. There is a glaring absence of any sort of progression to more robust, precise, and accurate functional models;
2. Articulation of an integrated, functional bio-psychosocial model is yet to occur with the field still being some distances from understanding how neurological and biological findings can be reliably understood in terms of daily psychological and social functioning; and
3. Years of research, with the aim of discovering the mechanisms of psychotherapeutic change, has not removed the uncertainty around why psychotherapy works for some and not for others. In the 10 years since Kazdin made this observation, we have made little progress addressing this crucial topic (3).

These conclusions can help to consider a problem from the perspective of whether it falls into one of three categories: simple; complicated; or complex (46). Our review would suggest that the problem of identifying therapeutic mechanisms is a complex problem. This is in keeping with conceptualizing psychotherapeutic mechanisms as part of a complex system (33). Complex problems encompass both simple and complicated problems within, but are not reducible to these problems (47). Solving a complex problem requires the solution to account for the special requirements and unique local conditions related to that problem (48), while allowing for parts of the problem to be interdependent (49), and not assuming linear causality (50). Addressing a complex problem requires one to understand that the problem will adapt given a change in conditions (51, 52). The field of research appears to have overlooked such requirements and, hence, has attempted to solve a complex problem using methods and means that are unsuited to the task; a criticism being echoed elsewhere (33, 39). Given Kant’s insight that our human powers of observation have built within them natural limitations that are designed to draw us toward inductive, causal interpretations which is an observation that is now further supported by more recent models of brain science and theories of reasoning, it might not be entirely unpredictable that the field of psychotherapy research would have reached the impasse it has (53–55). To find the elusive mechanisms being sought, for increased understanding of psychotherapeutic effectiveness, the field needs to move away from the ever growing “cobweb of learning” (56), toward functional mechanisms that can be falsified through the use of theory that acknowledges the complexity of the problem and processes related to that problem. Falsification in this sense would entail building simulation models of the proposed mechanisms that are capable of generating data. Then, the data from the model can be compared with the data produced by the suggested mechanism and the degree to which the data match will determine the acceptance or rejection of the mechanism.

## Conclusion

We argue that, for psychotherapy to improve its effectiveness and efficiency, the standard for what is accepted as a useful mechanism needs to be raised substantially. Only functional mechanisms that express plausible actions consistent with known biological processes should be used to inform therapeutic interventions. The current state of the evidence shows that the science has some distance to progress before we can be certain of the functional mechanisms underpinning psychotherapies.

## Author Contributions

TC conceived of the concept of the paper and prepared the initial submission as well as the first draft of the paper. SH conducted the searches for the review and led the review of the papers. All authors contributed to the review of the papers. RG and JD contributed substantially to writing and editing drafts of the paper to produce the final manuscript.

## Conflict of Interest

The authors declare that the research was conducted in the absence of any commercial or financial relationships that could be construed as a potential conflict of interest.
