# Supplementary material for: Identifying Functional Mechanisms in Psychotherapy: A Scoping Systematic Review
Source: Front Psychiatry. 2020 Apr 17;11:291. doi: 10.3389/fpsyt.2020.00291 (PMC7180170; doi:10.3389/fpsyt.2020.00291)
Supplement: Supplementary file 1 [file DataSheet_1.docx]

**Appendix A**

**Excluded studies**

Abreu PR, Hubner MMC, Lucchese F. The role of shaping the client’s interpretations in functional analytic psychotherapy. Analysis of Verbal Behavior. 2012;28:151–7.

**Reason for exclusion: Not research**

Babl A, Grosse Holtforth M, Perry JC, Schneider N, Dommann E, Heer S, et al. Comparison and change of defense mechanisms over the course of psychotherapy in patients with depression or anxiety disorder: Evidence from a randomized controlled trial. Journal of Affective Disorders. 2019;252:212–20.

**Reason for exclusion: Not functional mechanisms**

Babl A, Grosse Holtforth M, Heer S, Lin M, Stahli A, Holstein D, et al. Psychotherapy integration under scrutiny: Investigating the impact of integrating emotion-focused components into a CBT-based approach: A study protocol of a randomized controlled trial. BMC Psychiatry. 2016;16(1).

**Reason for exclusion: Study protocol only**

Beaulac J, Sadeh E. Can an 8-week psychotherapy group work for an early psychosis patient population? Yes and here’s how. Early Intervention in Psychiatry. 2018;12:198.

**Reason for exclusion: Not functional mechanisms**

Beissner F, Preibisch C, Schweizer-Arau A, Popovici RM, Meissner K. Psychotherapy With Somatosensory Stimulation for Endometriosis-Associated Pain: The Role of the Anterior Hippocampus. Biological Psychiatry. 2017;84(10):734–42.

**Reason for exclusion: Not functional mechanisms**

Beresford TP. Clinical assessment of psychological adaptive mechanisms in medical settings. Journal of Clinical Psychology. 2014;70(5):466–77.

**Reason for exclusion: Not research**

Boeker H, Kraehenmann R. Neuropsychodynamic approach to depression: Integrating resting state dysfunctions of the brain and disturbed self-related processes. Frontiers in Human Neuroscience. 2018;12.

**Reason for exclusion: Not research**

Brambilla F, Amianto F, Dalle Grave R, Fassino S. Lack of efficacy of psychological and pharmacological treatments of disorders of eating behavior: Neurobiological background. BMC Psychiatry. 2014;14(1).

**Reason for exclusion: Not functional mechanisms**

Brune M. On aims and methods of psychiatry-A reminiscence of 50 years of Tinbergen’s famous questions about the biology of behavior. BMC Psychiatry. 2014;14.

**Reason for exclusion: Not research**

Brunnhuber S. Differential-diagnosis of obsessive-compulsive symptoms in the borderline personality disorder. American Journal of Psychotherapy. 2003;57(4):460–70.

**Reason for exclusion: Not research**

Buckman JF, Vaschillo EG, Fonoberova M, Mezic I, Bates ME. The Translational Value of Psychophysiology Methods and Mechanisms: Multilevel, Dynamic, Personalized. Journal of Studies on Alcohol & Drugs. 2018;79(2):229–38.

**Reason for exclusion: Not research**

Busch AM, Callaghan GM, Kanter JW, Baruch DE, Weeks C. The functional analytic psychotherapy rating scale: A replication and extension. Journal of Contemporary Psychotherapy: On the Cutting Edge of Modern Developments in Psychotherapy. 2010;40(1):11–9.

**Reason for exclusion: Not functional mechanisms**

Busch AM, Kanter JW, Callaghan GM, Baruch DE, Weeks CE, Berlin KS. A Micro-Process Analysis of Functional Analytic Psychotherapy’s Mechanism of Change. Behavior Therapy. 2009;40(3):280–90.

**Reason for exclusion: Not functional mechanisms**

Busch AM, Manos RC, Rusch LC, Bowe WM, Kanter JW. FAP and behavioral activation. The practice of functional analytic psychotherapy. 2010;65–81.

**Reason for exclusion: Not research**

Callaghan GM, Follette WC, Ruckstuhl LE, Linnerooth PJN. The Functional Analytic Psychotherapy Rating Scale (FAPRS): A behavioral psychotherapy coding system. The Behavior Analyst Today. 2008;9(1):98–116.

**Reason for exclusion: Not research**

Callaghan GM, Summers CJ, Weidman M. The Treatment of Histrionic and Narcissistic Personality Disorder Behaviors: A Single-Subject Demonstration of Clinical Improvement Using Functional Analytic Psychotherapy. Journal of Contemporary Psychotherapy: On the Cutting Edge of Modern Developments in Psychotherapy. 2003;33(4):321–39.

**Reason for exclusion: Not functional mechanisms**

Callaghan GM. Functional Analytic Psychotherapy and supervision. International Journal of Behavioral Consultation and Therapy. 2006;2(3):416–31.

**Reason for exclusion: Not research**

Carey TA. Exposure and reorganization: The what and how of effective psychotherapy. Clinical Psychology Review. 2011;31(2):236–48.

**Reason for exclusion: Not research**

Carvalho MRD, Freire RC, Nardi AE. Virtual reality as a mechanism for exposure therapy. World Journal of Biological Psychiatry. 2010;11(2 PART 2):220–30.

**Reason for exclusion: Not research**

Centonze D, Siracusano A, Calabresi P, Bernardi G. Removing pathogenic memories: a neurobiology of psychotherapy. Molecular Neurobiology. 2005;32(2):123–32.

**Reason for exclusion: Not research**

Choi SW, Chi SE, Chung SY, Kim JW, Ahn CY, Kim HT. Is alpha wave neurofeedback effective with randomized clinical trials in depression? A pilot study. Neuropsychobiology. 2011;63(1):43–51.

**Reason for exclusion: Not functional mechanisms**

Clarkin JF, Cain NM, Lenzenweger MF. Advances in transference-focused psychotherapy derived from the study of borderline personality disorder: clinical insights with a focus on mechanism. Current Opinion in Psychology. 2018;21:80–5.

**Reason for exclusion: Not research**

Corrigan FM. Psychotherapy as assisted homeostasis: Activation of emotional processing mediated by the anterior cingulate cortex. Medical Hypotheses. 2004;63(6):968–73.

**Reason for exclusion: Not research**

De Meulemeester C, Vansteelandt K, Luyten P, Lowyck B. Mentalizing as a mechanism of change in the treatment of patients with borderline personality disorder: A parallel process growth modeling approach. Personality Disorders: Theory, Research, & Treatment. 2018;9(1):22–9.

**Reason for exclusion: Not functional mechanisms**

Dunlop BW, Hill E, Johnson BN, Klein DN, Gelenberg AJ, Rothbaum BO, et al. Mediators of sexual functioning and marital quality in chronically depressed adults with and without a history of childhood sexual abuse. Journal of Sexual Medicine. 2015;12(3):813–23.

**Reason for exclusion: Not functional mechanisms**

Ecker B, Toomey B. Depotentiation of symptom-producing implicit memory in coherence therapy. Journal of constructivist Psychology. 2008;21(2):87–150.

**Reason for exclusion: Not research**

Euler S, Stalujanis E, Allenbach G, Kolly S, De Roten Y, Despland JN, et al. Dialectical behavior therapy skills training affects defense mechanisms in borderline personality disorder: An integrative approach of mechanisms in psychotherapy. Psychotherapy Research. 2018;1–12.

**Reason for exclusion: Not functional mechanisms**

Feeny NC, Zoellner LA, Kahana SY. Providing a treatment rationale for PTSD: Does what we say matter? Behaviour Research and Therapy. 2009;47(9):752–60.

**Reason for exclusion: Not functional mechanisms**

Fisher H, Atzil-Slonim D, Bar-Kalifa E, Rafaeli E, Peri T. Growth curves of clients’ emotional experience and their association with emotion regulation and symptoms. Psychotherapy research : journal of the Society for Psychotherapy Research. 2019;29(4):463–78.

**Reason for exclusion: Not functional mechanisms**

Flor H. Lost in translation: Psychological mechanisms and psychotherapy. Lost in Translation: Psychologische Mechanismen und Psychotherapie. 2015;25(2):111–7.

**Reason for exclusion: Not research**

Flor H. Psychological pain interventions and neurophysiology: implications for a mechanism-based approach. American Psychologist. 2014;69(2):188–96.

**Reason for exclusion: Not research**

Fonzo G, Goodkind M, Oathes D, Zaiko Y, Harvey M, Peng K, et al. Reorganization of resting connectivity patterns following psychotherapy for posttraumatic stress disorder. Neuropsychopharmacology. 2017;43:S122–3.

**Reason for exclusion: Not research**

Fonzo GA, Goodkind MS, Oathes DJ, Zaiko YV, Harvey M, Peng KK, et al. Selective effects of psychotherapy on frontopolar cortical function in PTSD. American Journal of Psychiatry. 2017;174(12):1175–84.

**Reason for exclusion: Not functional mechanisms**

Fosha D. “Nothing that feels bad is ever the last step:” The role of positive emotions in experiential work with difficult emotional experiences. Clinical Psychology and Psychotherapy. 2004;11(1):30–43.

**Reason for exclusion: Not research**

Frye CG, Wardle MC, Norman GJ, De Wit H. MDMA decreases the effects of simulated social rejection. Pharmacology Biochemistry and Behavior. 2014;117:1–6.

**Reason for exclusion: Drug study**

Gaillard J-M. Neurobiological correlates of the unlocking of the unconscious. International Journal of Intensive Short-Term Dynamic Psychotherapy. 2000;14(4):89–107.

**Reason for exclusion: Not research**

Gallese V, Eagle MN, Migone P. Intentional attunement: Mirror neurons and the neural underpinnings of interpersonal relations. Journal of the American Psychoanalytic Association. 2007;55(1):131–76.

**Reason for exclusion: Not research**

Garratt G, Ingram RE, Rand KL, Sawalani G, Practice. Cognitive processes in cognitive therapy: Evaluation of the mechanisms of change in the treatment of depression. Clinical Psychology: Science. 2007;14(3):224–39.

**Reason for exclusion: Not research**

Goldbeck L, Fidika A, Herle M, Quittner AL. Psychological interventions for individuals with cystic fibrosis and their families. Cochrane Database of Systematic Reviews. 2014;(6):CD003148.

**Reason for exclusion: Systematic review**

Groves SJ, Porter RJ, Jordan J, Knight R, Carter JD, McIntosh VV, et al. Changes in neuropsychological function after treatment with metacognitive therapy or cognitive behavior therapy for depression. Depression & Anxiety. 2015;32(6):437–44.

**Reason for exclusion: Not functional mechanisms**

Gullestad FS, Johansen MS, Høglend P, Karterud S, Wilberg T. Mentalization as a moderator of treatment effects: findings from a randomized clinical trial for personality disorders. Psychotherapy research : journal of the Society for Psychotherapy Research. 2013;23(6):674–89.

**Reason for exclusion: Not functional mechanisms**

Gullestad FS, Wilberg T. Change in reflective functioning during psychotherapy--a single-case study. Psychotherapy Research. 2011;21(1):97–111.

**Reason for exclusion: Not functional mechanisms**

Hawro T, Lehmann S, Maurer M, Metz M. Anxiety about body symptoms increases perception of itch. Experimental Dermatology. 2015;24(3):E31.

**Reason for exclusion: Not functional mechanisms**

Herpertz SC, Schneider I, Schmahl C, Bertsch K. Neurobiological Mechanisms Mediating Emotion Dysregulation as Targets of Change in Borderline Personality Disorder. Psychopathology. 2018;51(2):96–104.

**Reason for exclusion: Not research**

Higginson S, Mansell W, Wood AM. An integrative mechanistic account of psychological distress, therapeutic change and recovery: The Perceptual Control Theory approach. Clinical Psychology Review. 2011;31(2):249–59.

**Reason for exclusion: Not research**

Higginson S, Mansell W. What is the mechanism of psychological change? A qualitative analysis of six individuals who experienced personal change and recovery. Psychology and Psychotherapy: Theory, Research and Practice. 2008;81(3):309–28.

**Reason for exclusion: Not quantitative research**

Hinshaw SP. Journal of Pediatric Psychology. Moderators and mediators of treatment outcome for youth with ADHD: Understanding for whom and how interventions work. 2007;32(6):664–75.

**Reason for exclusion: Not functional mechanisms**

Hofmann SG, Asmundson GJ, Beck AT. Behavior Therapy. The science of cognitive therapy. 2013;44(2):199–212.

**Reason for exclusion: Not research**

Houck JM, Moyers TB. Within-session communication patterns predict alcohol treatment outcomes. Drug and Alcohol Dependence. 2015;157:205–9.

**Reason for exclusion: Not functional mechanisms**

Huang X, Huang P, Li D, Zhang Y, Wang T, Mu J, et al. Early brain changes associated with psychotherapy in major depressive disorder revealed by resting-state fMRI: Evidence for the top-down regulation theory. International Journal of Psychophysiology. 2014;94(3):437–44.

**Reason for exclusion: Not functional mechanisms**

Johansson P, Høglend P. Clinical Psychology and Psychotherapy. Identifying mechanisms of change in psychotherapy: Mediators of treatment outcome. 2007;14(1):1–9.

**Reason for exclusion: Not research**

Kan LY, Henderson CE, von Sternberg K, Wang W. Does change in alliance impact alcohol treatment outcomes? Substance abuse. 2014;35(1):37–44.

**Reason for exclusion: Not functional mechanisms**

Kanter JW, Manbeck KE, Kuczynski AM, Maitland DWM, Villas-Boas A, Reyes Ortega MA. A comprehensive review of research on functional analytic psychotherapy. Clinical Psychology Review. 2017;58:141–56.

**Reason for exclusion: Systematic review**

Kauer SD, Reid SC, Crooke AH, Khor A, Hearps SJ, Jorm AF, et al. Self-monitoring using mobile phones in the early stages of adolescent depression: randomized controlled trial. Journal of medical Internet research. 2012;14(3):e67.

**Reason for exclusion: Not functional mechanisms**

Kaye WH, Wagner A, Fudge JL, Paulus M. Neurocircuity of eating disorders. 2011. p. 37–57.

**Reason for exclusion: Not research**

Kazdin AE. The Annual Review of Clinical Psychology. Mediators and mechanisms of change in psychotherapy research. 2007;3:1–27.

**Reason for exclusion: Not research**

Kazdin AE. Evidence-based treatment and practice: new opportunities to bridge clinical research and practice, enhance the knowledge base, and improve patient care. American Psychologist. 2008;63(3):146.

**Reason for exclusion: Not research**

Kazdin AE. Understanding how and why psychotherapy leads to change. Psychotherapy Research. 2009;19(4–5):418–28.

**Reason for exclusion: Not research**

King AP, Block SR, Sripada RK, Rauch S, Giardino N, Favorite T, et al. Altered Default Mode Network (Dmn) Resting State Functional Connectivity Following a Mindfulness-Based Exposure Therapy for Posttraumatic Stress Disorder (Ptsd) in Combat Veterans of Afghanistan and Iraq. Depression & Anxiety. 2016;33(4):289–99.

**Reason for exclusion: Not functional mechanisms**

Kiselnikov AA. Dysfunctional brain system underlying speech readiness in stuttering. International Journal of Psychophysiology. 2010;77(3):310–1.

**Reason for exclusion: Not research**

Kraemer HC, Wilson GT, Fairburn CG, Agras WS. Mediators and moderators of treatment effects in randomized clinical trials. Archives of general psychiatry. 2002;59(10):877–83.

**Reason for exclusion: Not research**

Kramer U, Pascual-Leone A, Rohde KB, Sachse R. Emotional Processing, Interaction Process, and Outcome in Clarification-Oriented Psychotherapy for Personality Disorders: A Process-Outcome Analysis. Journal of Personality Disorders. 2016;30(3):373–94.

**Reason for exclusion: Not functional mechanisms**

Kuczynski AM, Kanter JW, Wetterneck CT, Olaz FO, Singh RS, Lee EB, et al. Measuring intimacy as a contextual behavioral process: Psychometric development and evaluation of the awareness, courage, and responsiveness scale. Journal of Contextual Behavioral Science. 2019.

**Reason for exclusion: Article withdrawn**

Kumsta R. The role of epigenetics for understanding mental health difficulties and its implications for psychotherapy research. Psychology & Psychotherapy: Theory, Research & Practice. 2019;92(2):190–207.

**Reason for exclusion: Not research**

Kuyken W, Watkins E, Holden E, White K, Taylor RS, Byford S, et al. How does mindfulness-based cognitive therapy work? 2010;48(11):1105–12.

**Reason for exclusion: Not functional mechanisms**

Ladd BO, Garcia TA, Anderson KG. A novel application in the study of client language: Change talk in substance-using adolescents during a simulation task. Alcoholism: Clinical and Experimental Research. 2016;40:272A.

**Reason for exclusion: Not functional mechanisms**

Landes SJ, Kanter JW, Weeks CE, Busch AM. The impact of the active components of functional analytic psychotherapy on idiographic target behaviors. Journal of Contextual Behavioral Science. 2013;2(1–2):49–57.

**Reason for exclusion: Not functional mechanisms**

Landin-Romero R, Moreno-Alcazar A, Pagani M, Amann BL. How Does Eye Movement Desensitization and Reprocessing Therapy Work? A Systematic Review on Suggested Mechanisms of Action. Frontiers in Psychology. 2018;9:1395.

**Reason for exclusion: Systematic review**

Lawson DM, Davis D, Brandon S. Treating complex trauma: critical interventions with adults who experienced ongoing trauma in childhood. Psychotherapy: Theory, Research, Practice, Training. 2013;50(3):331–5.

**Reason for exclusion: Not research**

Lee SE, Han Y, Park H. Neural Activations of Guided Imagery and Music in Negative Emotional Processing: A Functional MRI Study. Journal of Music Therapy. 2016;53(3):257–78.

**Reason for exclusion: Not functional mechanisms**

Levy KN, Beeney JE, Wasserman RH, Clarkin JF. Conflict begets conflict: executive control, mental state vacillations, and the therapeutic alliance in treatment of borderline personality disorder. Psychotherapy Research. 2010;20(4):413–22.

**Reason for exclusion: Not functional mechanisms**

Li H, Jin R, Yuan K, Zheng B, Zheng Z, Luo Y, et al. Effect of electro-acupuncture combined with psychological intervention on mental symptoms and P50 of auditory evoked potential in patients with internet addiction disorder. Journal of Traditional Chinese Medicine. 2017;37(1):43–8.

**Reason for exclusion: Not functional mechanisms**

Liu L, Yao YW, Li CR, Zhang JT, Xia CC, Lan J, et al. The Comorbidity Between Internet Gaming Disorder and Depression: Interrelationship and Neural Mechanisms. Frontiers in Psychiatry. Frontiers Research Foundation. 2018;9:154.

**Reason for exclusion: Not functional mechanisms**

Loonen AJ, Ivanova SA. Circuits Regulating Pleasure and Happiness-Mechanisms of Depression. Frontiers in Human Neuroscience. 2016;10:571.

**Reason for exclusion: Not research**

Lorenzo-Luaces L, German RE, DeRubeis RJ. Clinical Psychology Review. It’s complicated: The relation between cognitive change procedures, cognitive change, and symptom change in cognitive therapy for depression. 2014;41:3–15.

**Reason for exclusion: Systematic review**

Lueken U, Straube B, Maslowski N, Wittchen HU, Strohle A, Wittmann A, et al. What happens in the brain after successful vs. non-successful CBT treatment? A multicenter fMRI study on panic disorder with agoraphobia. European Neuropsychopharmacology. 2012;22:S366–7.

**Reason for exclusion: Not functional mechanisms**

Luoto S, Karlsson H, Krams I, Rantala MJ. Depression subtyping based on evolutionary psychiatry: From reactive short-term mood change to depression. Brain, Behavior, and Immunity. 2018;69:630.

**Reason for exclusion: Not research**

Lynch TR, Chapman AL, Rosenthal MZ, Kuo JR, Linehan MM. Journal of Clinical Psychology. Mechanisms of change in dialectical behavior therapy: Theoretical and empirical observations. 2006;62(4):459–80.

**Reason for exclusion: Not research**

Maitland DWM. Functional analytic psychotherapy compared to watchful waiting for enhancing social connectedness: A randomized clinical trial with a diagnosed sample. Dissertation Abstracts International: Section B: The Sciences and Engineering. 2016;77(2–B(E)).

**Reason for exclusion: Not functional mechanisms**

Mangabeira V, Kanter J, Del Prette G. Functional Analytic Psychotherapy (FAP): A review of publications from 1990 to 2010. Special Issue: Functional Analytic Psychotherapy. 2012;7(2–3):78–89.

**Reason for exclusion: Systematic review**

Many MM, Kronenberg ME, Dickson AB. Creating a 'nest' of emotional safety: Reflective supervision in a child/parent psychotherapy case. Infant Mental Health Journal. 2016;37(6):717–27.

**Reason for exclusion: Pediatric study**

Mehling WE, Wrubel J, Daubenmier JJ, Price CJ, Kerr CE, Silow T, et al. Body Awareness: a phenomenological inquiry into the common ground of mind-body therapies. Philosophy, ethics, and humanities in medicine : PEHM. 2011;6:6.

**Reason for exclusion: Not quantitative research**

Mela C. The therapeutic model of Group Analytic Psychotherapy in Brain’s Plasticity modification and expression, in patients with Cognitive and Psychiatric Disorders: A Hypothesis of Neuron-Immune-Analysis and Neuron-Immune-modulation. Psychiatria Danubina. 2017;29(Suppl 3):389–98.

**Reason for exclusion: Not functional mechanisms**

Messina I, Sambin M, Beschoner P, Viviani R. Changing views of emotion regulation and neurobiological models of the mechanism of action of psychotherapy. Cognitive, Affective & Behavioral Neuroscience. 2016;16(4):571–87.

**Reason for exclusion: Not functional mechanisms**

Meyer-Lindenberg A. Neural mechanisms of risk for psychiatric disorders. European Neuropsychopharmacology. 2012;22:S111.

**Reason for exclusion: Not research**

Miller PW, McGowan IW, Bergmann U, Farrell D, McLaughlin DF. Stochastic resonance as a proposed neurobiological model for Eye Movement Desensitization and Reprocessing (EMDR) therapy. Medical Hypotheses. 2018;121:106–11.

**Reason for exclusion: Not research**

Milrod B, Altemus M, Gross C, Busch F, Silver G, Christos P, et al. Adult separation anxiety in treatment nonresponders with anxiety disorders: Delineation of the syndrome and exploration of attachment-based psychotherapy and biomarkers. Comprehensive Psychiatry. 2016;66:139–45.

**Reason for exclusion: Not functional mechanisms**

Mishra JPN, Shekhawat PS, Khangarot YS. Psycho-physiology of meditation. Indian Journal of Physiology and Pharmacology. 2011;55(5):32–3.

**Reason for exclusion: Not research**

Moyers TB, Martin T, Christopher PJ, Houck JM, Tonigan JS, Amrhein PC. Client language as a mediator of motivational interviewing efficacy: Where is the evidence? Alcoholism: Clinical and Experimental Research. 2007;31(SUPPL. 3):40S–47S.

**Reason for exclusion: Not functional mechanisms**

Murphy R, Cooper Z, Hollon SD, Fairburn CG %J B research, therapy. How do psychological treatments work? Investigating mediators of change. 2009;47(1):1–5.

**Reason for exclusion: Not research**

Newman MG, Fisher AJ. Mediated moderation in combined cognitive behavioral therapy versus component treatments for generalized anxiety disorder. Journal of Consulting and Clinical Psychology. 2013;81(3):405–14.

**Reason for exclusion: Not functional mechanisms**

Nock MK %J AC, Experimental Research. Conceptual and design essentials for evaluating mechanisms of change. 2007;31:4s–12s.

**Reason for exclusion: Not research**

Nocon A, Hackmann K, Pfister H, Heldmann B, Rosenhagen M, Lucae S, et al. Biomarkers for the combined efficacy of cognitive behavioural therapy in antidepressant treatment in depression. European Neuropsychopharmacology. 2009;19:S427.

**Reason for exclusion: Not functional mechanisms**

Northoff G, Bermpohl F, Schoeneich F, Boeker H. How does our brain constitute defense mechanisms? First-person neuroscience and psychoanalysis. Psychotherapy and Psychosomatics. 2007;76(3):141–53.

**Reason for exclusion: Not research**

Novac A, Bota RG. Transprocessing: A proposed neurobiological mechanism of psychotherapeutic processing. Mental Illness. 2014;6(1):20–35.

**Reason for exclusion: Not research**

Oshiro CKB, Kanter J, Meyer SB. A single-case experimental demonstration of Functional Analytic Psychotherapy with two clients with severe interpersonal problems. Special Issue: Functional Analytic Psychotherapy. 2012;7(2–3):111–6.

**Reason for exclusion: Not functional mechanisms**

Pankey J. Functional Analytic Psychotherapy (FAP) for cluster B personality disorders: Creating meaning, mattering, and skills. Special Issue: Functional Analytic Psychotherapy. 2012;7(2–3):117–24.

**Reason for exclusion: Not research**

Panksepp J, Wright JS, Döbrössy MD, Schlaepfer TE, Coenen VA. Affective neuroscience strategies for understanding and treating depression: From preclinical models to three novel therapeutics. Clinical Psychological Science. 2014;2(4):472–94.

**Reason for exclusion: Not research**

Parks-Sheiner AC. Positive psychotherapy: Building a model of empirically supported self-help. Dissertation Abstracts International: Section B: The Sciences and Engineering. 2009;70(6–B):3792.

**Reason for exclusion: Not functional mechanisms**

Pascual-Leone A. How clients “change emotion with emotion”: A programme of research on emotional processing. Psychotherapy research : journal of the Society for Psychotherapy Research. 2018;28(2):165–82.

**Reason for exclusion: Not research**

Potenza MN, Worhunsky PD, Kober H, Angarita G, Gallezot J, Matuskey D, et al. Dopaminergic function, choice impulsivity and cocaine use in cocaine-dependent humans. European Neuropsychopharmacology. 2015;25:S131–2.

**Reason for exclusion: Not functional mechanisms**

de Quervain DJF, Margraf J. Glucocorticoids for the treatment of post-traumatic stress disorder and phobias: A novel therapeutic approach. European Journal of Pharmacology. 2008;583(2–3):365–71.

**Reason for exclusion: Drug study**

Ranieri F. Children who dare too much: Child psychotherapy and extreme risk seeking. Journal of Child Psychotherapy. 2014;40(2):173–86.

**Reason for exclusion: Pediatric paper**

Ren Z, Ruan Y, Zhao Q, Zhang W, Lai L, Jiang G. The neuropsychological mechanism of therapy in depression and anxiety disorder: A meta-analysis of functional neuroimaging studies. Acta Psychologica Sinica. 2017;49(10):1302–21.

**Reason for exclusion: Systematic review**

Rizvi S, Cyriac A, Rusjan P, Strafella A, Sproule BA, Giacobbe P, et al. Predictors of DBS response: The impact of anhedonia and dopamine. European Neuropsychopharmacology. 2014;24:S316–7.

**Reason for exclusion: Not functional mechanisms**

Rodrigues H, Figueira I, Lopes A, Gonca̧lves R, Mendlowicz MV, Coutinho ESF, et al. Does d-cycloserine enhance exposure therapy for anxiety disorders in humans? A meta-analysis. PLoS ONE. 2014;9(7).

**Reason for exclusion: Systematic review**

Rosen GM, Davison GC %J B modification. Psychology should list empirically supported principles of change (ESPs) and not credential trademarked therapies or other treatment packages. 2003;27(3):300–12.

**Reason for exclusion: Not research**

Rosenbaum D, Maier MJ, Hudak J, Metzger FG, Wells A, Fallgatter AJ, et al. Neurophysiological correlates of the attention training technique: A component study. NeuroImage Clinical. 2018;19:1018–24.

**Reason for exclusion: Not functional mechanisms**

Rosenfeld B, Cham H, Pessin H, Breitbart W. Why is Meaning-Centered Group Psychotherapy (MCGP) effective? Enhanced sense of meaning as the mechanism of change for advanced cancer patients. Psycho-Oncology. 2018;27(2):654–60.

**Reason for exclusion: Not functional mechanisms**

Rougemont-Bücking A, Grivel J. Risk perception and emotional coping: A pathway for behavioural addiction? European Addiction Research. 2014;20(2):49–58.

**Reason for exclusion: Not research**

Rousseau A, Belleville G. The mechanisms of action underlying the efficacy of psychological nightmare treatments: A systematic review and thematic analysis of discussed hypotheses. Sleep Medicine Reviews. 2018;39:122–33.

**Reason for exclusion: Systematic review**

Ruggiero GM, Spada MM, Caselli G, Sassaroli S. A historical and theoretical review of cognitive behavioral therapies: From structural self-knowledge to functional processes. Journal of Rational-Emotive & Cognitive-Behavior Therapy. 2018;36(4):378–403.

**Reason for exclusion: Not research**

Sakai Y, Kumano H, Nishikawa M, Sakano Y, Kaiya H, Imabayashi E, et al. Changes in cerebral glucose utilization in patients with panic disorder treated with cognitive–behavioral therapy. NeuroImage. 2006;33(1):218–26.

**Reason for exclusion: Not functional mechanisms**

Sartorius A, Henn FA. Deep brain stimulation of the lateral habenula in treatment resistant major depression. Medical Hypotheses. 69(6):1305–8.

**Reason for exclusion: Not research**

Schellekens MPJ, Tamagawa R, Labelle LE, Speca M, Stephen J, Drysdale E, et al. Mindfulness-Based Cancer Recovery (MBCR) versus Supportive Expressive Group Therapy (SET) for distressed breast cancer survivors: Evaluating mindfulness and social support as mediators. Journal of Behavioral Medicine. 2017;40(3):414–22.

**Reason for exclusion: Not functional mechanisms**

Scherpiet S, Herwig U, Opialla S, Scheerer H, Habermeyer V, Jancke L, et al. Reduced neural differentiation between self-referential cognitive and emotional processes in women with borderline personality disorder. Psychiatry Research. 2018;233(3):314–23.

**Reason for exclusion: Not functional mechanisms**

Schmidt ID, Pfeifer BJ, Strunk DR. Putting the Cognitive Back in Cognitive Therapy: Sustained Cognitive Change as a Mediator of In-Session Insights and Depressive Symptom Improvement. Journal of Consulting and Clinical Psychology. 2019;87(5):446–56.

**Reason for exclusion: Not functional mechanisms**

Schnell K, Herpertz SC. Emotion Regulation and Social Cognition as Functional Targets of Mechanism-Based Psychotherapy in Major Depression With Comorbid Personality Pathology. Journal of Personality Disorders. 2018;32(Supplement):12–35.

**Reason for exclusion: Not research**

Schore AN. Relational trauma and the developing right brain: an interface of psychoanalytic self psychology and neuroscience. Annals of the New York Academy of Sciences. 2009;1159:189–203.

**Reason for exclusion: Not research**

Schwabe L, Nader K, Pruessner JC %J B psychiatry. Reconsolidation of human memory: Brain mechanisms and clinical relevance. 2014;76(4):274–80.

**Reason for exclusion: Not research**

Seo H-J, Choi YH, Chung Y-A, Rho W, Chae J-H. Changes in cerebral blood flow after cognitive behavior therapy in patients with panic disorder: a SPECT study. Neuropsychiatric disease and treatment. 2014;10:661.

**Reason for exclusion: Not functional mechanisms**

Serber ER, Fava JL, Christon LM, Buxton AE, Goldberger JJ, Gold MR, et al. Positive Psychotherapy to Improve Autonomic Function and Mood in ICD Patients (PAM-ICD): Rationale and Design of an RCT Currently Underway. Pacing & Clinical Electrophysiology. 2016;39(5):458–70.

**Reason for exclusion: Not functional mechanisms**

Shedler J. The efficacy of psychodynamic psychotherapy. American Psychologist. 2010;65(2):98.

**Reason for exclusion: Not research**

Shridhar U. Management and prevention of negative emotions by introducing positive attitude awareness (PAA) technique in psychosomatic physical therapy. Physiotherapy (United Kingdom). 2015;101:eS1395.

**Reason for exclusion: Not functional mechanisms**

Simek J. “Split mind” and EEG gamma activity in schizophrenia. Activitas Nervosa Superior. 2014;56(4):140–4.

**Reason for exclusion: Not research**

Starrs CJ, Perry JC. Coping Action Patterns as Mechanisms of Change Across Psychotherapies: Three Case Examples of Personality Disorders With Recurrent Major Depression. Journal of Personality Disorders. 2018;32(Supplement):58–74.

**Reason for exclusion: Not functional mechanisms**

Straube B, Lueken U, Jansen A, Konrad C, Gloster AT, Gerlach AL, et al. Neural correlates of procedural variants in cognitive-behavioral therapy: a randomized, controlled multicenter FMRI study. Psychotherapy and Psychosomatics. 2014;83(4):222–33.

**Reason for exclusion: Not functional mechanisms**

Thakur ER, Holmes HJ, Lockhart NA, Carty JN, Ziadni MS, Doherty H, et al. Mediational analysis of emotional awareness and expression training for patients with irritable bowel syndrome: A randomized trial. Gastroenterology. 2016;150(4):S694.

**Reason for exclusion: Not functional mechanisms**

Thiruchselvam T, Dozois DJA, Bagby RM, Lobo DSS, Ravindran LN, Quilty LC. The role of outcome expectancy in therapeutic change across psychotherapy versus pharmacotherapy for depression. Journal of Affective Disorders. 2019;251:121–9.

**Reason for exclusion: Not functional mechanisms**

Tryon WW, Hoffman J, McKay D. Neural network models as explanatory frameworks of psychopathology and its treatment. Treatments for psychological problems and syndromes. 2017;452–79.

**Reason for exclusion: Not research**

Tschacher W, Ramseyer F. Time-series based analysis of integrative psychotherapy of psychosis. European Archives of Psychiatry and Clinical Neuroscience. 2011;261:S36.

**Reason for exclusion: Not functional mechanisms**

Tschacher W, Zorn P, Ramseyer F. Change mechanisms of Schema-centered group psychotherapy with personality disorder patients. PLoS ONE. 2012;7(6).

**Reason for exclusion: Not functional mechanisms**

Tschacher W, Zorn P. Modeling patterns of change in the treatment of personality disorders. European Psychiatry. 2012;27.

**Reason for exclusion: Not functional mechanisms**

Turnbull OH, Fotopoulou A, Solms M. Anosognosia as motivated unawareness: the “defence” hypothesis revisited. Cortex; a journal devoted to the study of the nervous system and behavior. 2014;61:18–29.

**Reason for exclusion: Not research**

Uliaszek AA, Hamdullahpur K, Chugani CD, Rashid T. Mechanisms of change in group therapy for treatment-seeking university students. Behaviour Research and Therapy. 2018;109:10–7.

**Reason for exclusion: Not functional mechanisms**

Uribe MER, López Ramírez EO, Mena IJ. Effect of the EMDR psychotherapeutic approach on emotional cognitive processing in patients with depression. Spanish Journal of Psychology. 2010;13(1):396–405.

**Reason for exclusion: Not functional mechanisms**

Vago DR. Mapping modalities of self-awareness in mindfulness practice: a potential mechanism for clarifying habits of mind. Annals of the New York Academy of Sciences. 2014;1307:28–42.

**Reason for exclusion: Not research**

Valero-Aguayo L, Ferro-Garcia R, Kohlenberg RT, Tsai M. Procesos de Cambio Terapeutico en la Psicoterapia Analitica Funcional. Therapeutic change processes in functional analytic psychotherapy. 2011;22(3):209–21.

**Reason for exclusion: Not research**

Van Hoof JJM. The origin of mentalisation and the treatment of personality disorders. South African Journal of Psychiatry. 2010;16(3):99.

**Reason for exclusion: Not research**

van Rooij SJ, Geuze E, Kennis M, Rademaker AR, Vink M. Neural correlates of inhibition and contextual cue processing related to treatment response in PTSD. Neuropsychopharmacology. 2015;40(3):667–75.

**Reason for exclusion: Not functional mechanisms**

Vermetten E, Lanius R, Bremner JD. Can brain mechanism in PTSD help direct treatment advances? Basic and Clinical Pharmacology and Toxicology. 2010;107(S1):67.

**Reason for exclusion: Not research**

Watt DF, Panksepp J. Depression: An evolutionarily conserved mechanism to terminate separation distress? A review of aminergic, peptidergic, and neural network perspectives. Neuropsychoanalysis. 2009;11(1):7–51.

**Reason for exclusion: Not research**

Weeks CE, Kanter JW, Bonow JT, Landes SJ, Busch AM. Translating the theoretical into practical: A logical framework of functional analytic psychotherapy interactions for research, training, and clinical purposes. Behavior Modification. 2012;36(1):87–119.

**Reason for exclusion: Not research**

Weeks CE. An analogue study of the mechanism of change in Functional Analytic Psychotherapy. Dissertation Abstracts International: Section B: The Sciences and Engineering. 2014;74(9–B(E)):No-Specified.

**Reason for exclusion: Not functional mechanisms**

Weidong W, Haisheng Z, Hui D. The latest development of low resistance thought imprint psychotherapy sleep-regulating technique of insomnia. Sleep Medicine. 2013;14:e148.

**Reason for exclusion: Not functional mechanisms**

Welling H. Transformative emotional sequence: Towards a common principle of change. Journal of Psychotherapy Integration. 2012;22(2):109.

**Reason for exclusion: Not research**

Westen D, Novotny CM, Thompson-Brenner H. The empirical status of empirically supported psychotherapies: assumptions, findings, and reporting in controlled clinical trials. Psychological Bulletin. 2004;130(4):631.

**Reason for exclusion: Systematic review**

Whelton W. Emotional processes in psychotherapy: Evidence across therapeutic modalities. Clinical Psychology Psychotherapy: An International Journal of TheoryPractice. 2004;11(1):58–71.

**Reason for exclusion: Not research**

White TP, Borgan F, Ralley O, Shergill SS. You looking at me?: Interpreting social cues in schizophrenia. Psychological Medicine. 2016;46(1):149–60.

**Reason for exclusion: Not functional mechanisms**

Windgassen S, Moss-Morris R, Chilcot J, Sibelli A, Goldsmith K, Chalder T. The journey between brain and gut: A systematic review of psychological mechanisms of treatment effect in irritable bowel syndrome. British Journal of Health Psychology. 2017;22(4):701–36.

**Reason for exclusion: Systematic review**

Yager J. Updating empathy. Psychiatry: Interpersonal and Biological Processes. 2015;78(2):134–40.

**Reason for exclusion: Not research**

Yang Y, Kircher T, Straube B. Neural correlates of cognitive behavioral therapy in panic disorder. Neurale korrelate von kognitiver verhaltenstherapie bei panikstorung. 2015;63(2):79–87.

**Reason for exclusion: Not research**

Yuan TF, Hoff R. Mirror neuron system based therapy for emotional disorders. Medical Hypotheses. 2008;71(5):722–6.

**Reason for exclusion: Not research**

Zhao W, Jiang F, Zhang Z, Zhang J, Ding Y, Ji X. Remote Ischemic Conditioning: A Novel Non-Invasive Approach to Prevent Post-Stroke Depression. Frontiers in aging neuroscience. 2017;9:270.

**Reason for exclusion: Not research**
